# TXNRD2 (rs35934224) CT genotype and primary open-angle glaucoma

**DOI:** 10.5935/0004-2749.202200105

**Published:** 2022

**Authors:** Rujittika Mungmunpuntipantip, Viroj Wiwanitkit

**Affiliations:** 1 Private Academic Consultant, Bangkok, Thailand; 2 Honorary professor, Dr DY Patil University, Pune, India

Dear Editor:

We would like to share ideas on “TXNRD2 (rs35934224) CT genotype as a possible protective marker for primary open-angle glaucoma in a Brazilian population^(1)^.” Tenório et al. concluded that “Our data suggest an association between *TXNRD2* gene polymorphism (rs35934224) with primary open-angle...”^(1)^. We agree that *TXNRD2* (rs35934224) CT genotype might or might not be associated with primary open-angle glaucoma. It is also necessary to recognize the effects of possible confounding factors. It is necessary to realize the possible effect of other genetic polymorphisms that might be associated with primary open-angle glaucoma, although there is a control group in this report. Examples of those genetic polymorphisms comprise *CYP1B1* gene polymorphisms and variants in *MT-CYB* and *MT-ND4* genes^(2,3)^.
